# Open Surgical Conversion of Popliteal Endograft Infection: Case Reports and Literature Review

**DOI:** 10.3390/biomedicines12081855

**Published:** 2024-08-15

**Authors:** Marta Ascione, Ada Dajci, Rocco Cangiano, Antonio Marzano, Andrea Molinari, Francesca Miceli, Alessia Di Girolamo, Cristiana Leanza, Alessandra Oliva, Luca Di Marzo, Wassim Mansour

**Affiliations:** 1 Vascular and Endovascular Surgery Division, Department of General Surgery and Surgical Specialties, Policlinico Umberto I, “Sapienza” University of Rome, Viale del Policlinico 155, 00161 Rome, Italy; ada.dajci@uniroma1.it (A.D.); rocco.cangiano@uniroma1.it (R.C.); antonio.marzano@uniroma1.it (A.M.); andrea.molinari@uniroma1.it (A.M.); francesca.miceli@uniroma1.it (F.M.); alessia.digirolamo@uniroma1.it (A.D.G.); luca.dimarzo@uniroma1.it (L.D.M.); 2 Department of Public Health and Infectious Diseases, Sapienza University of Rome, 00185 Rome, Italy; cristiana.leanza@uniroma1.it (C.L.); alessandra.oliva@uniroma1.it (A.O.)

**Keywords:** popliteal aneurysm, endograft infection, popliteal endograft infection, open surgical conversion

## Abstract

Background: Endovascular treatment of popliteal aneurysms (PA) has increased in the last few years, quickly becoming the main treatment performed in many vascular centers, based on the acceptable and promising outcomes reported in the literature. However, endograft infections after endovascular popliteal aneurysm repair (EPAR) are the most dangerous complications to occur as they involve serious local compromise and usually require open surgical conversion and device explantation to preserve the affected extremity. Case report: We report two patients who were admitted to the emergency room of our hospital for pain and edema in the lower leg. Both patients had undergone exclusion of a ruptured PA a few years before by endovascular graft. CTA testing showed a significant volume of fluid-corpuscular collection related to perianeurysmal abscess collection in both cases. Blood cultures and drained material cultures were positive for *Staphylococcus capitis* in the first case and *S. aureus* in the second. Prophylactic antibiotics were administered for 10 days, then patients underwent an open surgical conversion with the complete explantation of endovascular material and a femoro-popliteal bypass using an autologous vein in the first case and a biological bovine pericardium prosthesis in the second case. The infective department of our hospital had defined a discharged specific antibiotic therapy for each patient, based on intraoperative microbiological samples. Furthermore, we have examined the literature and found six more cases described in case report articles that refer to popliteal graft infections by different microorganisms, mostly presenting acute limb ischemia as the first symptom and suggesting endograft explantation with open conversion and autologous vein bypass as the commonest therapeutic choice. Conclusions: The open surgical conversion of popliteal endograft infection is the best strategy to manage peripheral infection after an endovascular popliteal aneurysm repair procedure.

## 1. Introduction

Peripheral aneurismal diseases are rare and are encountered in less than 1% of the general population. However, the popliteal artery aneurysm (PAA) is the most common type, accounting for 70% of all peripheral arterial aneurysms. Its epidemiology is relevant to gender prevalence, with 95% in the male population, and it also presents anatomical links to its association with contralateral popliteal aneurysms in 20% of cases, with abdominal aortic aneurysms in 6.1% of cases [1,2]. The etiology processes have not been identified as yet. Some molecular studies suggest a combination of genetic and inflammatory factors. A decrease in the mechanical strength of the arterial wall, associated with an infiltration of inflammatory cells, appears to be implicated in aneurysm formation. Most PAA patients are asymptomatic at the time of detection [3], with an increasing need for intervention within 2–3 years in around 68% of cases [1]. The treatment of PAAs has evolved considerably over the years, but there is equipoise and a lack of consensus about the comparative effectiveness of either approach in managing PAAs. The traditional treatment approach is open surgery with aneurysm ligation or an exclusion bypass with an autologous vein or prosthetic conduit. However, the use of endovascular devices (endoprostheses and stents) has increased in the last few years, quickly becoming one of the primary treatments employed for these peripheral diseases. In 2022, the Society for Vascular Surgery published clinical practice guidelines on popliteal artery aneurysms [1] but it does not provide evidence of the superiority of open PAA repair (OPAR) versus endovascular PAA repair (EPAR), although all recent literature underlines the stackable outcomes of these invasive approaches. The importance of choosing an appropriate treatment lies in the clinical significance of PAA with the potentially limb-threatening sequelae that may arise, from acute limb ischemia to major amputation [4]. Thromboembolism for an aneurysmal sac that has thrombosed and a PAA that has ruptured are the first native complications of this limb disease, defining the major cause of occurrence in both the hospital and emergency treatment settings of PAA. Instead, the postoperative complications of both OPAR and EPAR could define a more severe setting with peripheral artery compromise, insufficient collateral vessels, or diffuse arterial disease that can be a precursor to severe peripheral limb impairment and a clear possibility of limb loss.

In this context, infective complications are identified as the rarest postoperative complications, but when infection occurs, it is burdened with high morbidity and mortality rates [5]. Late-onset infections may even present years after the initial procedure and symptoms are often vague [6]. This delayed and non-specific clinical presentation considerably affects the outcomes because misdiagnosis or delay in treatment are frequent. Management strategies depending on presentation severity, anastomotic graft involvement, stent graft involvement, and infectious microorganisms could include long-term antibiotic therapy, along with maximal invasive surgery with open conversion. Infected complications are well-described in the literature, reaching a rate of occurrence of 2.6% of all OPAR bypasses in the last few years [7,8,9], supported by Staphylococcus aureus infection [10]. However, infective EPAR complications are also very rare, with researchers identifying only six cases since 1980 [6,11].

The primary objective of this study is the evaluation of infectious complications related to endovascular implants in these aneurysmal diseases through a comprehensive review of the literature and by reporting two cases from our vascular unit experience. Furthermore, considering the severity of endograft infections, the desired aim is to find the best treatment strategy to save the limb as well as the patient’s life.

We decided to consider only the infectious complications of popliteal endografts after EPAR, excluding those infections related to all other popliteal disorders, such as occlusive disease.

## 2. Case Reports

### 2.1. Case A

**Figure 1:**
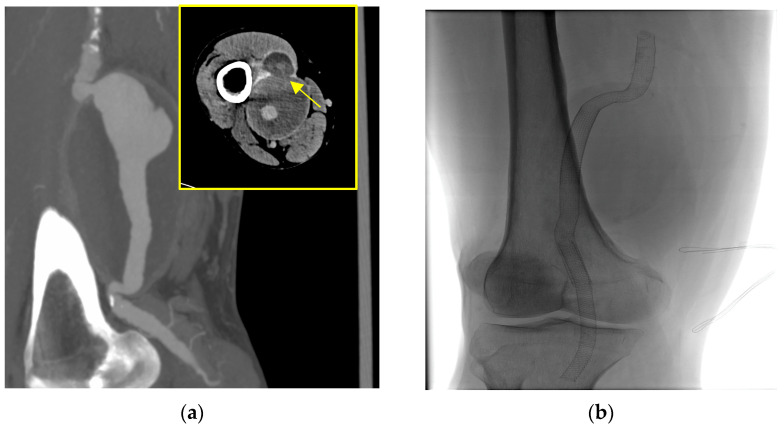

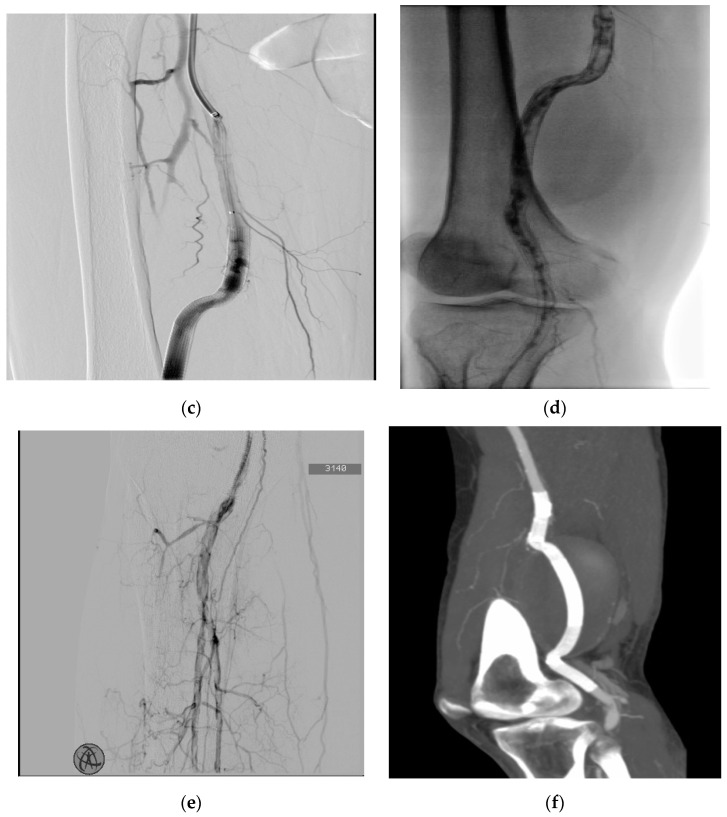
(**a**) Preoperative CTA, showing the ruptured PAA in the lateral projection and a focal rupture in the rectus femoris muscle in the axial projection in the yellow box marked by narrowing. (**b**) Final intraoperative radiographic control showing the VIABAHN™ endoprosthesis and Supera stent in the popliteal artery after the first endovascular emergency procedure. (**c**) An intraoperative angiography through the catheter shows proximal and (**d**) distal intrastent thrombosis. (**e**) Intraoperative complication with thrombosis of the anterior tibial artery and peroneal trunk. (**f**) Computer tomography angiography shows a perianeurysmal collection of approximately 9 cm and endovascular bypass patency.

**Figure 2:**
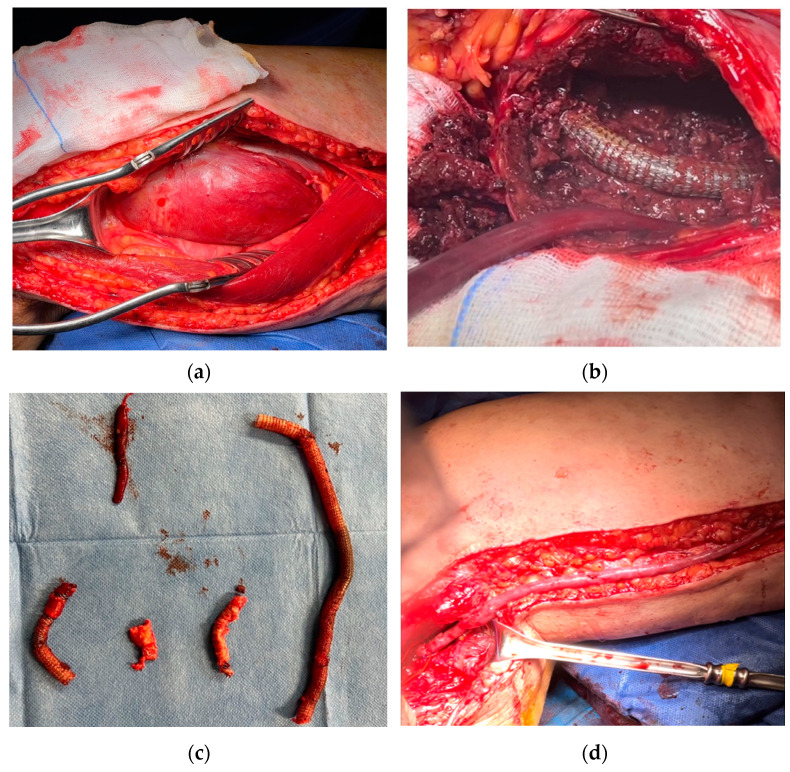
(**a**) Intraoperative section of medial tight access, revealing the wall of an intact aneurysmal collection. (**b**) Infected material (VIABAHN™ endoprosthesis) in the opened sac, with the infected graft lying inside. (**c**) Intraoperative stent graft: explanted materials. (**d**) Femoropopliteal bypass with the autologous reversed vein.

A 58-year-old man was admitted to the emergency room (ER) due to right lower extremity pain and pallor, involving the right leg, with motility and sensitivity reduced. An urgent CTA scan revealed a ruptured right PAA with poor below-the-knee (BTK) run-off vessels (Figure 1a). The patient underwent emergent hybrid treatment with a popliteal artery and distal vessel surgical embolectomy/thrombectomy using a Fogarty^©^ catheter, followed by an endovascular procedure with PAA exclusion using a VIABAHN™ endoprosthesis and Supera stent for the distal landing zone (Figure 1b). After two years, the patient complained of severe claudication; an ultrasound exam revealed severe intrastent restenosis (Figure 1c). The patient underwent an endovascular procedure with percutaneous transluminal angioplasty (PTA), using a drug-eluting balloon in an intraoperative complication with distal vessel thrombosis (Figure 1d), which was solved by thromboaspiration and intra-arterial thrombolysis treatment with urokinase for 48 h. The postoperative image examinations recorded a good recovery of artery patency, and the patient was discharged on the fourth postoperative day. Five days later, the patient was re-admitted to the hospital for acute right inferior limb ischemia. Again, a conservative approach was done using 24 h intra-arterial thrombolysis and two adjunctive VIABAHN™ endoprosthesis was implanted because of disconnection between the VIABAHN™ that had previously been implanted. After two months, the patient returned to the hospital for the third time, presenting pain, hyperemia, and swelling of the right leg. A CT scan showed a fluid-corpuscular collection surrounding the endoprosthesis, which was related to the aneurysmal abscess collection of the right superficial femoral (SFA) and popliteal arteries (Figure 1e). The patient was admitted to our department to investigate the local popliteal abscess. Working with the infectious disease department, we took three sets of haemocultures, which tested positive for Staphylococcus capitis. We then performed local drainage with a puncture in the proximal area of the abscess and it revealed Staphylococcus capitis infection. We discussed our case with a multidisciplinary team, and we felt it was appropriate to start antibiotic therapy with daptomycin and then proceed to open surgery. After two weeks of prophylactic antibiotic therapy, the patient underwent a surgical conversion with the complete explantation of endovascular material (Figure 2b,c), and we performed a femoropopliteal bypass using an autologous vein (Figure 2d), with good patency of the downstream vessels after surgery.

Antibiotic therapy was continued until a negative haemoculture result was obtained and no fever episode occurred. In addition, the infectious disease team had deemed appropriate the choice of an antibiotic discharge therapy (trimethoprim/sulfamethoxazole), suggested from discharge to the control visit after one month. They directly referred the patient to their ambulatory unit, and, after one month, no fever and no other clinical suspected symptoms were reported.

One year after the procedure, the vascular ultrasound follow-up documented good patency of the bypass and the downstream vessel.

### 2.2. Case B

A 76-year-old man was admitted to the ER for fever and swelling on the left leg. Physical examination of the left lower limb showed a hyperemic and painful area from the third distal thigh to the ankle. In the medical history, the patient underwent an endovascular intervention in an emergent setting to exclude a left ruptured popliteal aneurysm with a VIABAHN™ endoprosthesis. The patient declared a recent visit to another hospital with the same fever symptoms a few months before, with a sepsis status and positive hemoculture for Staphylococcus aureus. The patient was treated with antibiotic therapy and, after three weeks, was discharged.

**Figure 3:**
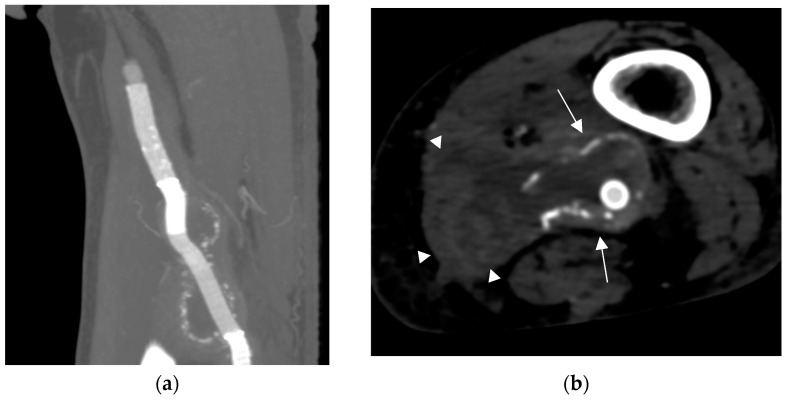
(**a**) CTA images showing the popliteal collection surrounding the popliteal stent graft; (**b**) CTA images showing the popliteal endograft (arrows) and the ruptured collection in the femoral muscles (arrowheads).

CTA was performed and showed a peri-aneurysmatic fluid-corpuscular collection around the left popliteal artery (Figure 3). Working with the infectiology department of our institution, we collected three sets of haemocultures and performed local drainage via a puncture of the popliteal abscess: all samples tested positive for Staphylococcus aureus. A multidisciplinary team with an infective consultant recommended joint antibiotic therapy and open surgical treatment by abscess drainage and popliteal region reclamation, performing explantation of the infected endovascular devices and a femoropopliteal bypass. After two weeks of antibiotic therapy, the patient underwent the surgical procedure; we decided to use a biological pericardium bovine prosthesis for the femoropopliteal bypass because of the poor quality of the patient’s saphenous veins. The final complete perviousness of the downstream vessels is acceptable.

The Infectiology team had decided to continue antibiotic therapy until a negative hemoculture was achieved and no fever episode occurred. Moreover, they chose to administer a single dalbavancin dose before the discharge because of multiple patient comorbidities (a previous coronary and a carotid stent). Again, they referred this second patient to the infective ambulatory unit; after one month, normal-range blood test results were recorded, and no other clinically suspect symptoms were noted.

The one-year follow-up revealed the ultrasound patency of the bypass and tibiofemoral vessels and complete remission from infective status.

## 3. Material and Methods

This study reports a comprehensive review of the literature over the last 50 years regarding cases of late-onset popliteal endograft infection. Data about the risk factors, clinical presentation, medical and surgical treatment, outcomes, and mortality were collected and compared.

### 3.1. Literature Review

**Figure 4:**
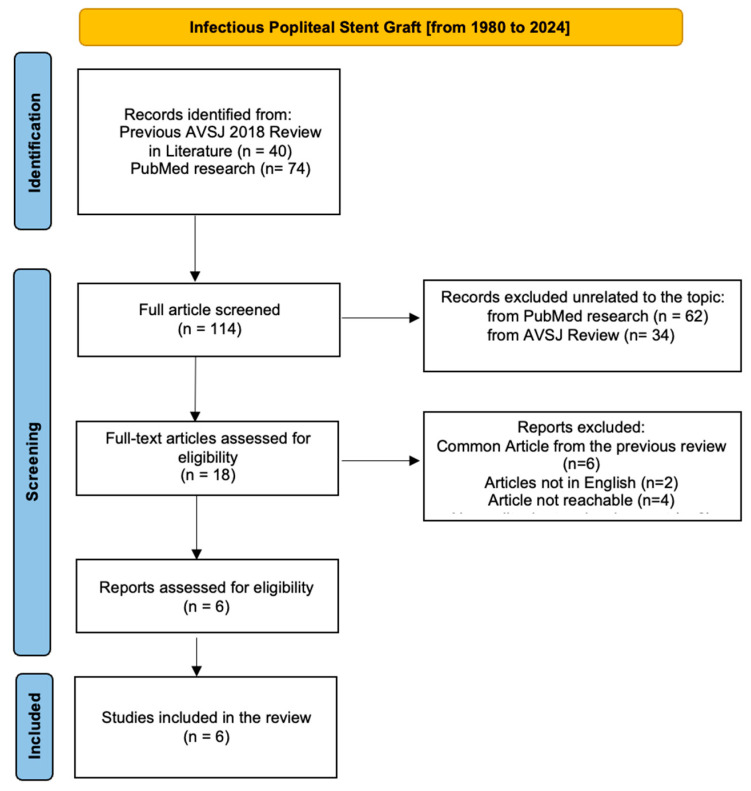
The PRISMA chart identifies 114 articles as free PubMed research: of these, 6 full papers adequately described a popliteal infected stent graft.

One author of this article conducted a comprehensive review of the literature from 1980 to 2024 through the preferred reporting items for systematic review (PRISMA) procedure to collect data about reported cases of late-onset infections over bare-metal or covered popliteal artery stents. The search terms were: [stent infection] AND [peripheral stent infection] AND [infectious popliteal stent graft] OR [infective popliteal stent graft]. We identified 114 articles as free PubMed research. Firstly, the titles and abstracts were reviewed for corrected appropriacy related to graft infection in endovascular procedures; then, we searched and deeply analyzed all articles identifying a graft infection in the popliteal artery related to the endovascular procedure. As we underline in the PRISMA chart (Figure 4), we excluded those articles without an available full text or that were missing in an English international language version. Subsequently, we analyzed all articles more closely to exclude those regarding all graft infections not involving the popliteal artery. We did not consider a limited time frame in the analysis in order to define all popliteal stent graft infections from the beginning of the endovascular procedure.

We deeply analyzed a 2018 review in the literature that was published by Whitcher et al. [11], presenting a comprehensive literature review of all peripheral vascular stent graft infections published in volume 51 of the Annals of Vascular Surgery Journal (AVSJ). This complete review analyzed infective complication vascular-stent grafts in all peripheral arteries reported from 1980 to 2018. Also, Bosman et al. [5] published a case report and review of the literature about infections of intravascular bare-metal stents in 2013 [5], but we deemed it appropriate to consider the AVSJ review as being more recent, complete, and exhaustive. Moreover, all popliteal infection EJVS-cited articles are included in the AVSJ review (Figure 4).

The author extracted data from each study using a predefined database form summarized in Table 1 and included the following information: general data (author name, year, and type of study) and clinical data (risk factors, clinical presentation, imaging findings, responsible bacteria, medical and surgical treatment, complications, and outcomes). The extracted data are reported as percentages and absolute values (Table 2 and Table 3) [11].

### 3.2. Results

We identified around 114 articles from the PubMed research [11,12,13,14,15] and we chose 6 papers as being most relevant. Of these six articles, five are case reports [11,12,13,14,16], and one deals with a coup d’oeil [15]. The Gharacholou paper [17] was considered not to be pertinent to the field because it described an endovascular procedure for occlusive disease of the popliteal artery instead of an aneurysm [17].

Finally, our review of the literature from 1980 to 2024 included six articles, mostly case reports, referring to specific popliteal stent graft infections. Table 1 describes and compares the principal data. We also considered our two cases in the final results, resulting in data from eight total patients with popliteal endograft infection.

**Table 1:** General information about the review of the literature. The articles were dated from 1980 to 2024 [16].

Author	Year	Study Type	Gender	Stent Type	Onset Infective Symptom Timeline
Giannoukas et al. *[13]*	1999	Case report	Male	BMS (Strecker stent) *	16 days
Green et al. *[12]*	2013	Case report	Female	BMS (Bard) *	3 weeks
Houthoofd et al. *[16]*	2012	Case report	Male	Stent graft (Hemobahn)	24 months
Walker et al. *[15]*	2017	Coup d’oeil	Male	Endoprosthesis (VIABAHN™)	12 months
Witcher et al. *[11]*	2018	Case report and review in literature	Female	BMS (Bard)*	72 months
Macheda et al. *[14]*	2003	Case report	Male	BMS (Palmaz, Cordis) *	18 months
*Our center*	2024	Case report (x2)	Male	Stent graft (VIABAHN™)	2 months; 24 months

* BMS: bare-metal stent.

**Table 2:** Clinical presentation and the responsible microorganism. More than one clinical issue could be presented in the same patient.

**Clinical Presentation**	**Number**	**%**
Acute limb ischemia	6	75
Skin ulceration/skin erythema	5	62
Distal necrosis/gangrene/petechiae	2	25
Claudication	1	12
Pseudoaneurysm (CT images)	1	12
Abscess/peri-arterial infiltration/fluid collection (CT images)	5	62
**Microorganism**	**Number**	**%**
Gram-positive (*Staphylococcus aureus*)	4	20
Gram-positive (*Staphylococcus epidermidis*)	1	12
Gram-positive (*Staphylococcus capitis*)	1	12
Gram-negative (*Proteus mirabilis*)	1	12
Gram-negative (*Pseudomonas aeruginosa*)	1	12

**Table 3:** Treatment and outcomes.

**Treatment**	**Number**	**%**
Autologous vein bypass	4	62
Prosthesis bypass	2	25
Tied artery	1	12
Conservative management	1	12
**Outcomes**	**Number**	**%**
Major amputation	1	12
Minor amputation	2	37
Death	0	
Good downstream vessel flow after 1 month	7	87
Claudication > 100 mt	1	16

Eight patients were included in our literature database referring to stent graft infection involving only the popliteal artery. The studied population was mostly composed of males (six males and two females). The main onset symptoms were acute limb ischemia in six cases and different skin manifestations from erythema (four cases) to distal gangrene in two cases. The main instrumental report was of CTA abscesses, documented as a periarterial collection in five cases. The main risk factors recorded were the same as in the vascular population: hypertension (six patients) and diabetes mellitus (two patients); there was either no reference to genetic disease (Marfan syndrome or Ehlers–Danlos syndrome), or immunological disease was found. Laboratory analysis and instrumental analysis were not available in almost all cases. Two cases clearly demonstrated a high elevation of white blood cells and the inflammatory index (active C-protein).

The screening of the updated literature revealed the effectiveness of open surgery treatment in seven cases, mostly using an autologous vein bypass in four of eight cases; two patients underwent a prosthetic bypass, one with a ring stripper [16] and one with a pericardium bovine prosthesis; only one instance of conservative management was reported, due to patient intervention denial. The outcomes clarify the effectiveness of autologous vein bypass interventions and document the patency of the bypass with good downstream vessel flow at one year after the surgery. Minor amputation (mostly toe amputation) was judged necessary to preserve the distal lower limb and prevent other possible causes of infection. Only one patient underwent major amputation (below-the-knee amputation) because the lower limb was judged to be un-recoverable, with surgery preventing massive toxic system damage due to acute prolonged limb ischemia.

## 4. Discussion

Endovascular stent infection is a rare but potentially lethal complication of interventional vascular procedures. It first involves the surrounding stent area, causing local symptoms (pain, edema, and swelling), but it can quickly become a systemic infection, compromising and causing the failing of multiple organs, and leading to death. Therefore, it is very important to diagnose and eradicate infection as quickly as possible [18]. Several studies involving both OPAR and EPAR define a compromised local setting with a clear possibility of limb loss. In particular, few infective complications after OPAR have been described in case reports in the last few years [7,8,9]; however, a recent JVS paper of the Vascular and Endovascular Surgery Division of Harvard Medical School [10] describes a 25-year experience of infective OPAR bypass, with 34 cases in 1315 interventions defying the risk of bypass graft infection (BGI) at around 2.6%, mostly caused by Staphylococcus aureus. As we underline in our literature review section, the infective EPAR complication is also very rare, with the search identifying only six cases after 1980 [6,11].

The timeline of onset symptoms could suggest the principal causes of this terrible complication and, as already described in our review of the literature, they are wide-ranging. Stent infection mainly occurs during the first two weeks and up to two months after placement, suggesting probable contamination due to inadequate sterile techniques. Late-onset symptoms could suggest an independent correlation with the primary vascular procedure that is probably related to other urological procedures, dental work, or general infections. It could also take into account the various patients and their different co-morbidities to understand the different rates of infection and the higher likelihood of developing an infectious disease. As we already discussed in Section 3.2, most diagnoses of an infected stent may be clinically suggested by septic distal emboli causing acute limb ischemia or other manifestations such as skin erythema, petechiae, or distal gangrene. Instrumental diagnosis methods are also fundamental to identifying, quantifying, and defining other organ involvement, thereby stratifying surgical risk. In the CTA images, the infection is shown in peri-stent soft tissue inflammation as fluid collection. It could also be found as pseudoaneurysm formation at a stent site, even though this is a rare condition (in our review of the literature, only two patients out of eight exhibited it).

Moreover, microbiological samples are fundamental to understanding the supporting infective mechanism. The different microbiological samples could monitor the infective state, with positive haemocultures and abscess drainage samples suggesting a systemic compromise. Blood tests could also offer a simple and clear but not specific systemic infective monitoring index. The most common organisms implicated in vascular graft infections are Gram-positive and Gram-negative bacteria, such as Staphylococcus and Pseudomonas [5,6], and this finding is also supported by our review of the literature, indicating the same incidence of popliteal infection and general vascular infection, also after an open procedure [10]. Microorganism identification is a fundamental step for the management of the disease because, through the right antibiotic choice, the main attack route can be identified, and this could influence all patients’ outcomes. Above all, considering that infective stent complications are fatal, it seems to be a priority to identify the causative bacteria in the international management of these popliteal stent graft infections.

We believe that the best approach for the definitive treatment of an infected stent requires multidisciplinary team discussions to finalize the best antibiotic support for successful open surgery conversion. We believe that the final approach to an infected endovascular device is to explant it and drain the infected area to create a new, safe bypass. To achieve the best outcome rates, even in emergency settings, vascular surgeons must perform open surgical conversion in the safest setting possible, choosing the right antibiotic therapy to protect the patient, treating the infected area, and choosing the most appropriate time for the surgical intervention. Thanks to the infectiology department of our institution, we believe that a combination of prophylactic and post-operative antibiotic therapy and open surgical conversion is the best management strategy to eradicate the infection, with drainage of the local infectious area, and to prevent possible systemic re-infection. In our hospital’s cases, our infectiology team participated in every step of the disease management. They set up the same empiric antibiotic therapy (piperacillin/tazobactam + daptomycin) for both patients, from their appearance in the ER to the microorganism identification. Then, they started personalized antibiotic therapy with daptomycin; in both cases, this was two weeks before surgery. The multidisciplinary team identified the best surgical approach as open surgery, considering it more efficacious and powerful for the drainage of local infectious areas. The surgical conversion was performed two weeks after empiric therapy started and one week after microbiological identification based on abscess sampling, followed by specific antibiotic therapy. The choice of bypass conduit was based on the patency and adequacy of the autologous vein but, when the vein tributary was considered poor (as in our second reported case), there were valuable different solutions, as Wu et al. described [19]. Instead, in our case, the team preferred a pericardium bovine tube graft because it provided acceptable mid- and long-term results in native or prosthetic vascular infections, as described in several reports [20,21].

After the surgical conversion, patients continued antibiotic therapy until a negative hemoculture result was obtained and no fever episode occurred. Moreover, the infectiology team deemed it appropriate to choose a different discharge antibiotic therapy. In the first case, the choice of antibiotic discharge therapy (trimethoprim/sulfamethoxazole) was suggested for the period from the discharge until the control visit after one month; in the second case, they chose a single dalbavancin dose before discharge because of multiple patient comorbidities (a previous coronary and a carotid stent). Both patients were referred to the infective ambulatory unit and, after one month, they exhibited normal-range blood test results, with no fever, and no other clinical suspected symptoms were recorded.

During the infectiological follow-up, both patients underwent chronic antibiotic therapy using Dalbavancin until the PET-CTA scan results were negative.

**Figure 5:**
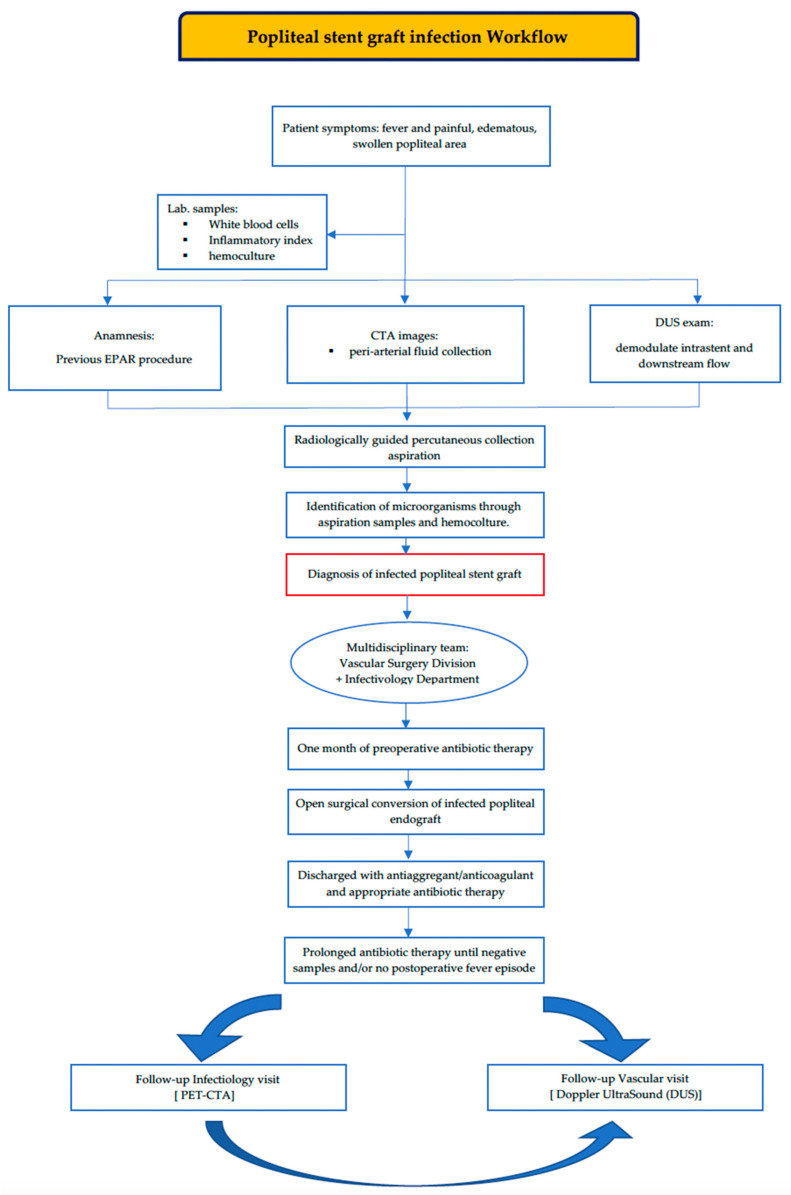
Workflow of popliteal stent graft infection, as developed in our center. EPAR: endovascular PAA repair; CTA: computer tomography angiography; DUS: ultrasound exam; PET: positron emission tomography.

However, the vascular follow-up visit conducted after one year documented good patency bypass and downstream vessels, confirming that there was no fever episode or other clinical symptoms during the period of a year after the surgery. Our workflow is shown to more deeply understand our multidisciplinary decision process (Figure 5).

### Limitations

There are several limitations to this study. Firstly, the literature review is based on databases that rely solely on accurate site reporting, but these have limited power due to the small sample size. Therefore, it is possible that the investigators did not identify all popliteal graft infections. Also, infection sampling methods have improved over the years, and this could imply important issues: an underestimated sample and identification of disastrous cases of popliteal graft infection with the most complicated management. However, we have to emphasize a double time limitation related to the management of the graft infection and the follow-up. The analysis of microbiological specimens and antibiotic management have changed a lot over the years and there is no international management as yet. Also, discussion about the mid–early and late complications of endovascular procedures has arisen in recent years, with the first prospective data on follow-up. Future refinements should analyze graft infection complications using internationally accepted methods, both in the samples and in the peri- and post-operative management of graft infection. This will be one of the major issues addressed in the discussion of endovascular procedures in the coming years.

## 5. Conclusions

Popliteal stent graft infection identifies a tragic post-operative complication, with several major sequelae to probably fatal exitus. It seems to be necessary to identify an international management of this disease to prevent systemic involvement and save patients’ lives. Supporting the literature review, this study suggests the fundamentals of a multidisciplinary team approach. The collaboration of vascular surgeons and the infectiology team is necessary for the treatment of lower limb infection disease through open surgery, as well as to prevent systemic infective involvement with appropriate antibiotic therapy. Even if our study is only supported by a limited number of patients, the open surgical conversion of popliteal endograft infection with a bypass conduit seems to be the best surgical strategy to manage peripheral infection of the popliteal stent graft.

In the future, it could be helpful to create an international register, aiming to identify the best worldwide approaches for these infectious peripherical aneurismal pathologies. The knowledge gained and the data collected will help to plan treatment strategies addressing and preventing several infectious diseases that are associated with the use of endovascular devices for the treatment of popliteal aneurysms on an international scale. We propose our multidisciplinary approach workflow as a start for future collaboration.

## Data Availability

The original contributions presented in the study are included in the article; further inquiries can be directed to the corresponding authors.
